# Characteristics of Damage to Brown Rice Kernels under Single and Continuous Mechanical Compression Conditions

**DOI:** 10.3390/foods13071069

**Published:** 2024-03-30

**Authors:** Xiaopeng Liu, Ziang Shi, Yonglin Zhang, Hui Li, Houchang Pei, Hongjun Yang

**Affiliations:** 1 School of Mechanical Engineering, Wuhan Polytechnic University, Wuhan 430023, China; 18772461221@163.com (Z.S.); yin248247640@163.com (Y.Z.); lihui09@whpu.edu.cn (H.L.); peihouchang@163.com (H.P.); hongjun_yang@163.com (H.Y.); 2 Hubei Cereals and Oils Machinery Engineering Center, Wuhan 430023, China

**Keywords:** brown rice kernels, rice milling, elastic–plastic contact, continuum damage, Hertz theory

## Abstract

During the rice milling process, single and continuous compression occurs between brown rice and the processing parts. When the external load exceeds the yield limit of brown rice, brown rice kernels are damaged; with an increase in compression deformation or the extent of compression, the amount of damage to the kernels expands and accumulates, ultimately leading to the fracture and breakage of kernels. In order to investigate the mechanical compression damage characteristics of brown rice kernels under real-world working conditions, this study constructs an elastic–plastic compression model and a continuous damage model of brown rice kernels based on Hertz theory and continuous damage theory; the accuracy of this model is verified through experiments, and the relevant processing critical parameters are calculated. In this study, three varieties of brown rice kernels are taken as the research object, and mechanical compression tests are carried out using a texture apparatus; finally, the test data are analysed and calculated by combining them with the theoretical model to obtain the relevant critical parameters of damage. The results of the single compression crushing test of brown rice kernels showed that the maximum destructive forces *F*_c_ in the single compression of Hunan Early indica 45, Hunan Glutinous 28, and Southern Japonica 518 kernels were 134.77 ± 11.20 N, 115.64 ± 4.35 N, and 115.84 ± 5.89 N, respectively; the maximum crushing deformations *α*_c_ in the single compression crushing test were 0.51 ± 0.04 mm, 0.43 ± 0.01 mm, and 0.48 ± 0.17 mm, respectively; and the critical average deformations *α*_s_ of elasticity–plasticity deformation were 0.224 mm, 0.267 mm, and 0.280 mm, respectively. The results of the continuous compression crushing test of brown rice kernels showed that the critical deformations *α*_d_ of successive compression damage formation were 0.224 mm, 0.267 mm, and 0.280 mm, and the deformation ratios *δ* of compression damage were 12.24%, 14.35%, and 12.84%. From the test results, it can be seen that the continuous application of compression load does not result in the crushing of kernels if the compression deformation is less than *α*_d_ during mechanical compression. The continuous application of compressive loads can lead to fragmentation of the kernels if the compressive deformation exceeds *α*_d_; the larger the compression variant, the less compression is required for crushing. If the compression deformation exceeds *α*_c_, then a single compressive load can directly fragment the kernels. Therefore, the load employed during rice milling should be based on the variety of brown rice used in order to prevent brown rice deformation, which should be less than *α*_d_, and the maximum load should not exceed *F*_c_. The results of this study provide a theoretical reference for the structure and parameter optimisation of a rice milling machine.

## 1. Introduction

**Figure 1:**
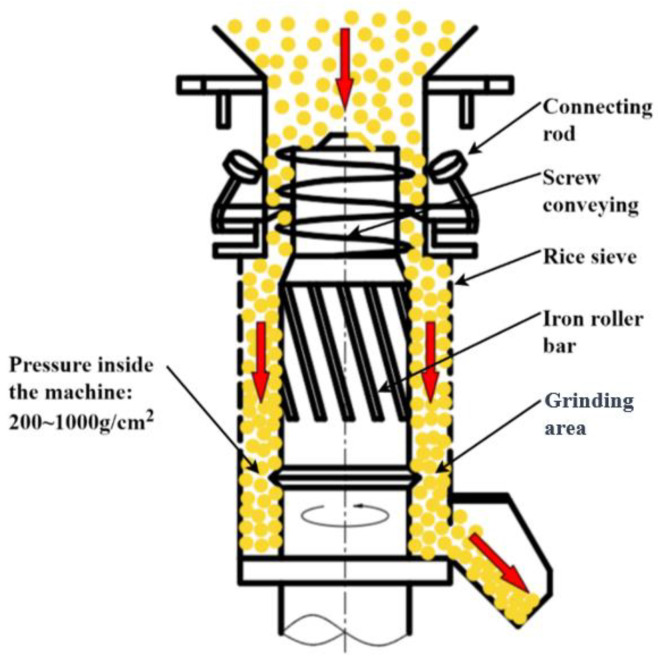
A wipe-away-type rice milling machine.

**Figure 2:**
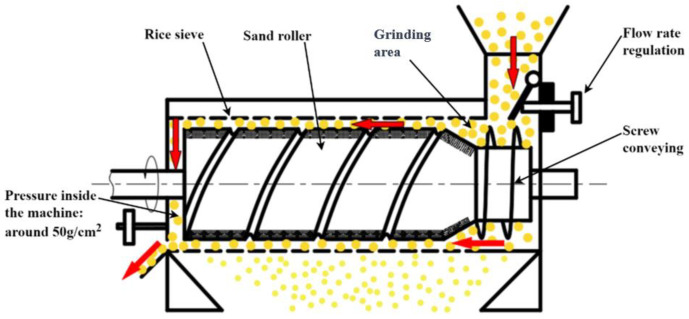
A grinding-type rice milling machine.

China is a large paddy-producing country, and as of 2023, the national rice production of China reached 206.6 billion kg. Paddy becomes rice after being processed by cleaning, hulling, grain and brown separation, milling, polishing, colour sorting, grading, and packing. Mechanical compression exists in the above processing, and paddy kernels are susceptible to damage and breakage due to mechanical compression, which ultimately leads to a reduction in both the yield and quality of the finished rice [1,2,3]. Research shows that when paddy is hulled to form brown rice, in the subsequent milling process, brown rice is prone to damage and can be broken, denoting this as the main source processing link of broken rice [4,5,6,7]. At present, both domestic and foreign rice milling production lines basically encompass the use of a rice milling machine for the mechanical milling of brown rice; the processing principle of the rice milling machine roller on brown rice is to produce extrusion and friction so that the milling chamber can peel off the skin of brown rice for it to become white rice. Both wipe-away-type and grinding-type rice milling machines are commonly used. In a wipe-away-type rice milling machine (Figure 1), the main processing parts of the iron roller, due to the iron roller surface friction coefficient being small, need the structural parameters of the equipment to be adjusted so that there is greater pressure in the milling chamber in order to meet the processing requirements. At this stage, brown rice is susceptible to damage and breakage due to a large single compression force. In a grinding-type rice milling machine (Figure 2), for the main processing parts of the sand roller, due to the sand roller surface friction coefficient being large, the milling chamber requires a smaller amount of pressure for the continuous grinding action to meet the processing requirements. However, during processing, a large number of brown rice kernels are squeezed against each other, and in addition to some brown rice kernels being broken due to single compression, more brown rice kernels are continuously compressed over a long period of time, resulting in the accumulation of damage, and breakage occurs when the accumulated damage exceeds the limit [8,9,10,11]. In summary, it can be seen that both single and continuous mechanical compression are important factors in the damage and breakage of brown rice during mechanical milling.

Numerous studies have been conducted on the mechanical compression properties of brown rice. The majority of existing research focuses on the analysis of influencing factors and the crushing process. Through their analysis, Sun Jingxin et al. determined that compression orientation had the greatest effect on grain compression characteristics, followed by moisture content, while variety had the least influence [12]. Zhou Xianqing et al. obtained the compression, bending, and shear mechanical properties of a variety of rice during mechanical processing, all of which were most heavily affected by compression force [13]. Feng Shuaibo et al. investigated the mechanism of crack generation and expansion during the mechanical compression of brown rice and showed that cracks were formed from the inside and expanded radially outwards from the endosperm centre [14]. Yang L et al. concluded that compressive loading is a key factor in the rupture of brown rice and pointed out that there are three stages to the crushing process: elastic deformation, plastic deformation, and mixed fracture through tests [15]. Fan Y et al. analysed the tribological behaviour of different varieties of brown rice using a self-developed friction prototype and pointed out that the wear mechanism of brown rice consists of fatigue damage, plastic deformation, adhesive wear, and abrasive wear [16]. The above research mainly utilised the single compression of brown rice kernels to carry out relevant tests and analyse the elastic–plastic phase in the crushing process, ignoring the continuous compression of brown rice in the actual milling process; they did not construct a complete theoretical model for the elastic–plastic phase in the crushing process of brown rice, so the results of the above research are of limited significance for the control of actual rice milling parameters.

In the study of the continuous damage characteristics of material kernels, the current research mainly focuses on exploring the continuous damage process and damage model of particles. Chen Yan et al. analysed the changing law of stiffness of lychee during continuous loading by testing [17]. Antonyuk S et al. used Hertzian theory to investigate the effects of grain size and stress velocity on fracture force and contact stiffness during elastic and elastoplastic deformation, in addition to the compressive behaviour of kernels under repeated loading and unloading conditions [18]. Russell, A. et al. presented key information on the deformation and fracture behaviour of elastoplastic kernels under quasi-static compressive forces from an energetic point of view, and they also analysed the strain hardening of kernels during local repetitive compression [19]. Dong XY et al. conducted an analytical and numerical study of the local contact loading–unloading behaviour of elastomers and elastic–ideal plastomers under continuous conditions [20]. Antonyuk S et al. developed a contact model for describing the compression behaviour of elastic–plastic kernels starting with the elastic compression behaviour of kernels described by the Hertz theory; they pointed out that under repeated loading with a constant load amplitude, the kernels show cyclic hardening, the coefficient of restitution increases, and plastic deformation reaches a certain degree of saturation [21]. From the above studies, it can be seen that the continuous damage process of material kernels is usually divided into the elastic deformation stage, plastic deformation stage, and cumulative damage stage, and there are differences in the continuous damage process and models of different kinds of materials due to differences in their shapes. At present, few studies have been conducted on the continuous damage characteristics of brown rice kernels, but ones which have referenced other grains such as maize and wheat provide some ideas. For example, Z Chen et al. tested the damage resistance of maize and wheat when subjected to repeated impacts and quantitatively obtained the damage resistance of both kernels under different continuous loading conditions [22]. However, on the whole, the current research on the compression characteristics of various types of grains mainly focuses on single compression performance, and research focusing on continuous damage characteristics is extremely limited.

Grinding-type rice milling machines are the mainstay of the current rice milling process. In order to solve the problem of the over-milling and crushing of brown rice due to the lack of a complete equipment structure and processing parameters under real-world working conditions, this paper details a study on the single and continuous compression damage characteristics of brown rice kernels. The elastic–plastic compression model and the continuum damage model of brown rice kernels were established to obtain the relevant damage critical parameters of brown rice kernels (maximum destructive force in single compression, *F*_c_; maximum crushing deformation in single compression, *α*_c_; critical average deformation of elasticity–plasticity deformation, *α*_s_; critical deformation of successive compression damage formation, *α*_d_; and deformation ratio of compression damage, *δ*). The mechanical damage mechanism of brown rice kernels under single and continuous compression conditions was investigated to provide a theoretical reference for the optimisation of the structure and parameters of grinding-type rice milling machines.

## 2. Analysis of Mechanical Compression Characteristics of Brown Rice Kernels

### 2.1. Hertz Contact Theory for Brown Rice Kernels

**Figure 3:**
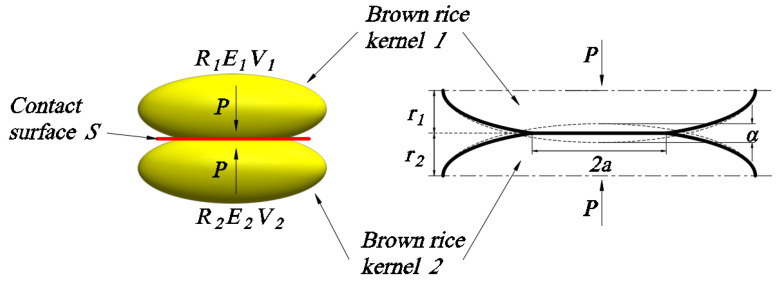
Hertz contact of brown rice kernels.

As shown in Figure 3, brown rice kernels can be approximated as ellipsoids, and combined with the Hertz theory [23,24], the normal stress distribution on the ellipsoidal contact surface is:

(1) $$P\left(x,y\right)=\frac{3F}{2\pi ab}\sqrt{1-\frac{x^{2}}{a^{2}}-\frac{y^{2}}{b^{2}}}$$

where *x* and *y* are the coordinates of the contact surface; the origin is at the centre of the contact surface; *F* is normal pressure; and *a* and *b* are the radii of the long and short axes of the elliptical contact surface, respectively.

From Equation (1), it can be seen that maximum normal stress is perpendicular to the origin of the contact surface, and its magnitude is as follows:

(2) $$P_{max}=\frac{3F}{2\pi ab}$$

The radii of the long and short axes of the elliptical contact surface *a*, *b* are as follows:

(3) $$a=m{\left[\frac{3FA}{2\left(\frac{1}{R_{1}}+\frac{1}{R_{1}^{\prime }}+\frac{1}{R_{2}}+\frac{1}{R_{2}^{\prime }}\right)}\right]}^{\frac{1}{3}}$$

(4) $$b=n{\left[\frac{3FA}{2\left(\frac{1}{R_{1}}+\frac{1}{R_{1}^{\prime }}+\frac{1}{R_{2}}+\frac{1}{R_{2}^{\prime }}\right)}\right]}^{\frac{1}{3}}$$

(5) $$A=\frac{1-\mu_{1}^{2}}{E_{1}}+\frac{1-\mu_{2}^{2}}{E_{2}}$$

where *m* and *n* are the contact coefficients of the two contacting objects; *R*_1_, $R_{1}^{\prime }$, *R*_2_, $R_{2}^{\prime }$ are the maximum and minimum radii of the curvature of the two contacting objects at the contact point; and *E*_1_, *E*_2_, *µ*_1_, *µ*_2_ are the modulus of elasticity and Poisson’s ratio of the two contacting objects, respectively.

The maximum and minimum radii of the curvature *R*, $R^{\prime }$ of the ellipsoid at the point of contact when an external load is applied along the height direction are, respectively:

(6) $$R=\frac{L^{2}}{2H}$$

(7) $$R^{\prime }=\frac{B^{2}}{2H}$$

where *L*, *B*, and *H* are the length, width, and thickness of the ellipsoid, respectively.

Based on the Hertz theory, the compressive displacement *α* between the ellipsoids along the load direction is as follows:

(8) $$\alpha ={k\left[\frac{9F^{2}A^{2}}{\pi^{2}}\left(\frac{1}{R_{1}}+\frac{1}{R_{1}^{\prime }}+\frac{1}{R_{2}}+\frac{1}{R_{2}^{\prime }}\right)\right]}^{\frac{1}{3}}$$

where *m*, *n*, and *k* can be calculated from the radii of the curvature *R*_1_, $R_{1}^{\prime }$, *R*_2_, $R_{2}^{\prime }$ at the point of contact between the two objects and the angle of direction *θ*:

(9) $$cost=\frac{{\left[{\left(\frac{1}{R_{1}}-\frac{1}{R_{1}^{\prime }}\right)}^{2}+{\left(\frac{1}{R_{2}}-\frac{1}{R_{2}^{\prime }}\right)}^{2}+2\left(\frac{1}{R_{1}}-\frac{1}{R_{1}^{\prime }}\right)\left(\frac{1}{R_{2}}-\frac{1}{R_{2}^{\prime }}\right)cos2\theta \right]}^{\frac{1}{2}}}{\frac{1}{R_{1}}+\frac{1}{R_{1}^{\prime }}+\frac{1}{R_{2}}+\frac{1}{R_{2}^{\prime }}}$$

The value of *cost* is known and the values of *m*, *n*, and *k* can be found from the table of exposure relationships in the ASAE S368.4 DEC2000 (R2008) standard [25].

### 2.2. Analysis of the Elastic Compression of Brown Rice Kernels

In this study, the Hertz contact theory was used to calculate the normal force on brown rice kernels during mechanical compression [26,27,28]. In order to ensure that the mechanical compression process of brown rice kernels satisfies the Hertz theory, it was assumed that brown rice kernels are isotropic bodies with smooth and homogeneous surfaces, and that elastic deformation is much smaller than the thickness of the kernels; there is no relative slippage between the contact surfaces, and the tangential force is zero at low-speed (loading speed of 0.02 mm/s) mechanical compression.

**Figure 4:**
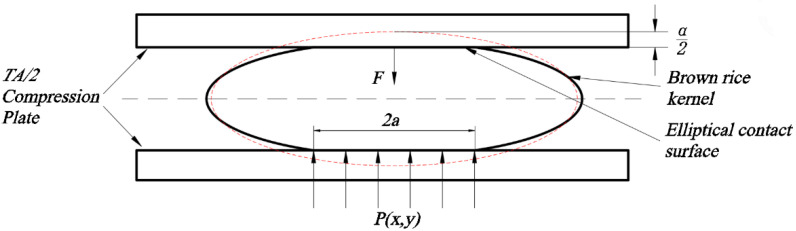
Elastic deformation of brown rice kernels.

As shown in Figure 4, the mechanical compression test of brown rice kernels was carried out using a TA/2 compression tool to apply the load, and the modulus of elasticity of brown rice kernels was much smaller than that of steel, which was used to modify Equation (5):

(10) $$A\approx \frac{1-\mu^{2}}{E}$$

where *E* and *µ* are the modulus of elasticity and Poisson’s ratio of brown rice kernels.

Brown rice kernels have an approximate ellipsoidal shape; when the flat plate is mechanically compressed along the normal direction (thickness), the maximum and minimum radii of the curvature of the contact points are, respectively:

(11) $$R_{1}=\frac{L^{2}}{2H},\;R_{1}^{\prime }=\frac{B^{2}}{2H}$$

(12) $$R_{2}=\infty ,\;R_{2}^{\prime }=\infty$$

where *R*_1_, $R_{1}^{\prime }$, *R*_2_, $R_{2}^{\prime }$ are the maximum and minimum radii of the curvature of the brown rice kernels and flat plate at the contact point; and *L*, *B*, and *H* are the length, width, and thickness of the brown rice kernels, respectively.

The mechanical compression equation for brown rice kernels can be obtained by incorporating Equations (10)–(12) into Equation (8):

(13) $$\;F=\frac{\pi }{3}k^{-\frac{3}{2}}E^{*}{R_{e}}^{\frac{1}{2}}\alpha^{\frac{3}{2}}$$

where E* is the integrated modulus of elasticity, which can be expressed as follows:

(14) $$\;E^{*}=\frac{E}{1-\mu^{2}}$$

and *R_e_* is the equivalent radius of brown rice kernels, which can be expressed as follows:

(15) $$R_{e}={\left(\frac{1}{R_{1}}+\frac{1}{R_{1}^{\prime }}\right)}^{-1}$$

Equations (11) and (12) can be obtained by incorporating them into Equation (9):

(16) $$cost=\frac{L^{2}-B^{2}}{L^{2}+B^{2}}$$

The *cost* value is known, and the required *k* value can be found from the contact relationship table in the ASAE S368.4 DEC2000 (R2008) standard [25].

From Equation (13), it can be seen that “*F-α* ^3/2^” denotes a linear relationship, the test can be obtained from the pressure and shape variable data, the least squares method is used to fit “*F-α* ^3/2^”, Poisson’s ratio *µ* is known, and, by substituting into Equation (13), the apparent contact elastic modulus *E* of brown rice can be obtained:

(17) $$E=\frac{F}{\alpha^{\frac{3}{2}}}\frac{3}{\pi }\left(1-\mu^{2}\right)k^{\frac{3}{2}}{R_{e}}^{-\frac{1}{2}}$$

### 2.3. Elastic–Plastic Compression Modelling of Brown Rice Kernels

From the relationship between the pressure and compression shape variable obtained from the single compression characteristic test of brown rice kernels, it can be seen that the *F-α* curve fits well with the Hertz theory curve before the intersection, and that the degree of deviation between the curves after the intersection increases gradually with the increase in the compression shape variable, meaning that the Hertz theory cannot accurately describe the whole process of the mechanical compression of brown rice kernels.

Brown rice kernels are nonhomogeneous materials by nature; when the compression load exceeds its yield limit, the microscopic defects inside the kernels will expand and merge and produce obvious plastic deformation at the contact surface, and from the *F-α* curves obtained from the tests, it can be seen that a more obvious linear reinforcement process exists in the mechanical compression of brown rice kernels [29,30]. In this study, the elastic–plastic compression model of brown rice kernels was constructed with reference to the theory of elastic–plastic reinforcement and subsequent experiments, which can better describe the whole process of the actual mechanical compression of brown rice kernels [31].

**Figure 5:**
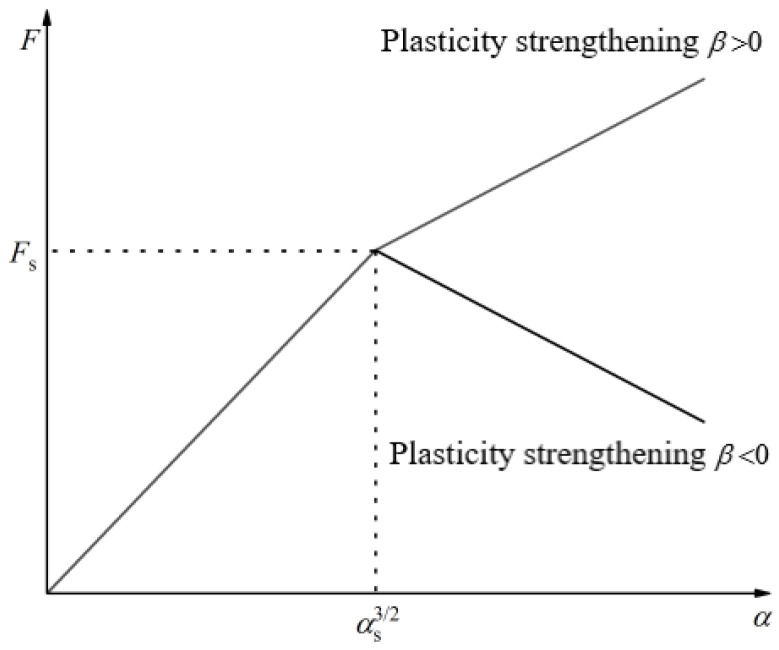
“*F*-*α*” at the point of contact of brown rice kernels.

**Figure 6:**
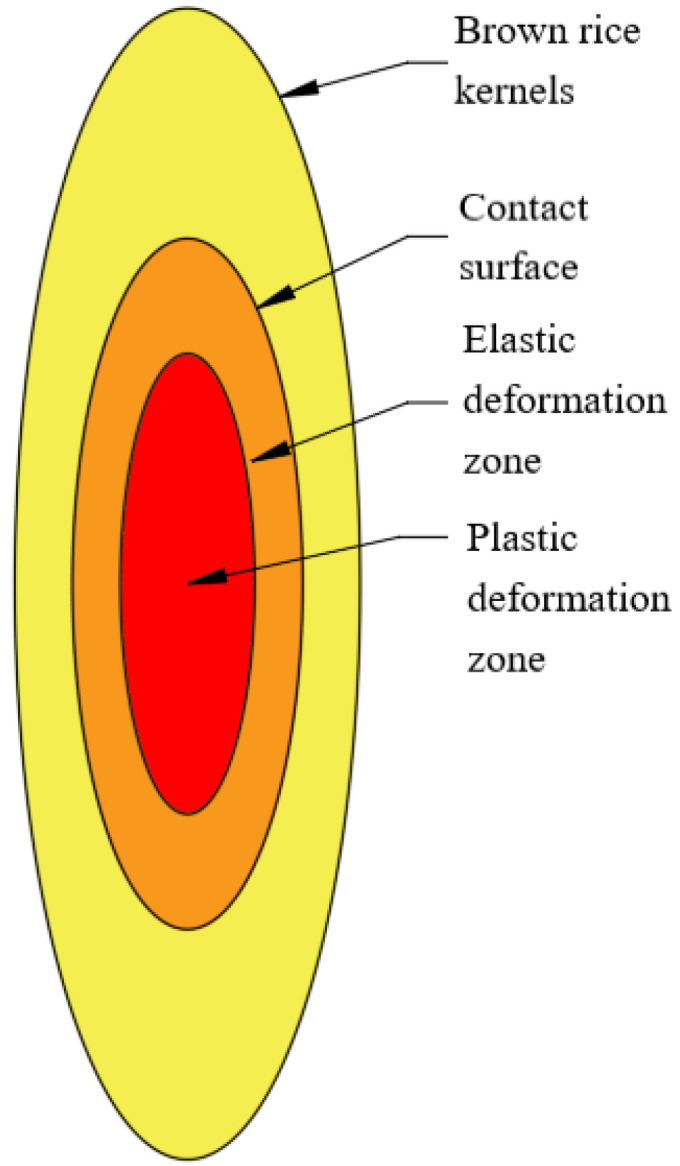
Contact zone.

From Equation (13), it can be seen that normal pressure is proportional to the 3/2 power of compression displacement, assuming that brown rice kernels satisfy the elastic–plastic linear strengthening relationship shown in Figure 5. When the external load reaches the yield limit of brown rice, the continuously increasing pressure produces a plastic deformation zone at the part of the contact surface that is weaker, as shown in Figure 6.

From Equations (2) and (13), it can be seen that critical yield stress corresponds to compressive displacement when the external load reaches the brown rice yield limit: *β*

(18) $$P_{s}=\frac{E^{*}{R_{e}}^{\frac{1}{2}}\alpha_{s}^{\frac{3}{2}}}{2ab}$$

Referring to the assumption that the material satisfies the von Mises yield criterion as suggested by Brizmer V et al. [32] based on the Hertz theory, yield stress is calculated as follows:

(19) $$P_{s}=\left(1.234+1.256\mu \right)Y$$

where *P*_s_ is critical yield stress and *Y* is yield strength.

From Equations (18) and (19), it can be seen that compressive displacement at theoretical critical yielding is given as follows:

(20) $$\alpha_{s}={\left[\frac{2abY\left(1.234+1.256\mu \right)}{E^{*}R_{e}^{\frac{1}{2}}}\right]}^{\frac{2}{3}}$$

The mathematical model of normal pressure *F* and compression displacement *α* during the elastoplastic compression of brown rice kernels is as follows:

(21) $$F=\left\{\begin{array}{c} \frac{\pi }{3}k^{-\frac{3}{2}}E^{*}R_{e}^{\frac{1}{2}}\alpha_{s}^{\frac{3}{2}}\;\;\;\;\;\;\alpha^{\frac{3}{2}}⩽\alpha_{s}^{\frac{3}{2}}\\ F_{s}+\beta \left(\alpha^{\frac{3}{2}}-\alpha_{s}^{\frac{3}{2}}\right)\;\;\;\alpha^{\frac{3}{2}}⩾\alpha_{s}^{\frac{3}{2}} \end{array}\right.$$

where *F*_s_ is the critical yield pressure; *α*_s_ is the compression displacement at this point; and *β* is the strengthening (softening) coefficient, the value of which is determined for brown rice kernels of a defined variety and moisture content.

### 2.4. Continuous Damage Model for Brown Rice Kernels

From the multiple compression characterisation tests of brown rice kernels, it is clear that the application of a continuous compression load does not result in the breakage of kernels when the compression load is less than the yield limit. When the compression load exceeds the yield limit when brown rice cracks, by applying a continuous compression load, irreversible damage will produce accumulation, expansion, and fusion; ultimately, the brown rice will be broken. In this study, a continuous damage model for brown rice kernels is constructed with reference to the continuous damage theory and subsequent experiments, which can better describe the continuous mechanical compression damage evolution process of brown rice kernels [33].

The continuous damage variable *D* for brown rice kernels is defined as follows:

(22) $$D=1-\frac{E^{\prime }\;}{E^{*}}\alpha \geq \alpha_{s}$$

where $E^{\prime }$ is the effective modulus of elasticity of brown rice kernels; E* is the combined modulus of elasticity of brown rice kernels; and *α*_s_ is the yield ultimate compression displacement.

**Figure 7:**
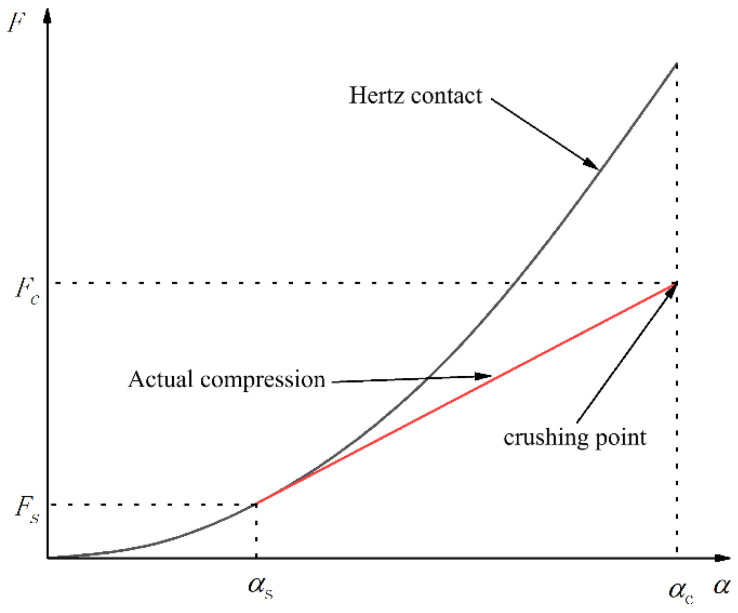
Compression force–deformation curve of brown rice kernels.

The process of producing complete damage in brown rice kernels is shown in Figure 7, and with reference to the damage model proposed by Tavares [34], the equation for the evolution of compression damage in brown rice kernels is derived as follows:

(23) $$D={\left(\frac{\alpha -\alpha_{s}}{\alpha_{c}-\alpha_{s}}\right)}^{\gamma }$$

where *α_c_* is the deformation of brown rice kernels when broken, and *γ* is the damage coefficient, the size of which is related to the material of the contacting object itself; the other symbols have the same meaning as previously outlined.

**Figure 8:**
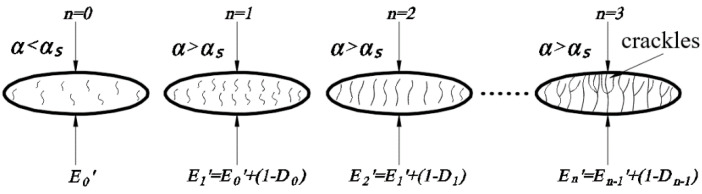
Evolution of continuous compression damage in brown rice kernels.

The essence of the damage is the change in the effective modulus of elasticity of the kernels. By combining Equation (22) and using the linear cumulative damage criterion to calculate the continuous compression damage accumulation of brown rice kernels, assuming that the number of compressions is *n*, the effective modulus of elasticity of brown rice kernels is as follows:

(24) $$E_{n}^{\prime }=E_{n-1}^{\prime }\left(1-D_{n-1}\right)=E^{\prime }\prod_{i=1}^{n-1}\left(1-D_{i}\right)$$

where the value of $E_{n-1}^{\prime }$ is the effective modulus of elasticity of brown rice kernels after the *n*-1st compression; and *D_n_*_-1_ is the amount of damage produced by the *n*-1st compression of brown rice kernels, as shown in Figure 8. 

Therefore, the relationship between normal pressure *F* during multiple brown rice compressions and the compression shape variable *α* is as follows:

(25) $$F=\left\{\begin{array}{c} \frac{\pi }{3}k^{-\frac{3}{2}}E^{*}{R_{e}}^{\frac{1}{2}}\alpha^{\frac{3}{2}}\;\;\;\;\;\;\;\;\;\alpha ⩽\alpha_{s}\\ F_{s}+E^{\prime }\prod_{i=1}^{n-1}\;\left(1-D_{i}\right)R_{e}^{\frac{1}{2}}\left(\delta^{\frac{3}{2}}-\delta_{d}^{\frac{3}{2}}\right)\;\;\;\alpha ⩾\alpha_{s} \end{array}\right.$$

## 3. Materials and Methods

In order to verify the accuracy of the elastic–plastic compression model of brown rice kernels and the continuous damage model of brown rice kernels, firstly, the basic physical parameters of brown rice were measured, and then a single compression test of brown rice kernels was carried out; images of the whole process from the compression to the crushing of the kernels were recorded during the test, and the results were verified and the relevant characteristics were calculated after the test was completed. The actual compression shape variable displacement curves, the theoretical curves of Hertz contact, the elastic–plastic curves, and the consistency of the actual compression curves with the theoretical curves were plotted and analysed. Finally, the continuous compression test of brown rice kernels was carried out, and the crack extension images of the kernels were recorded at the end of each compression during the test. After the test, the continuous compression and crushing data were summarised, the damage limit intervals were marked out and verified, the variation curves of the continuous compression force with the deformation variable were plotted, and the characteristic parameters of the different compressions were calculated in order to analyse the continuous damage process and to verify the consistency between the actual damage evolution process and the theoretical linear damage cumulative criterion.

### 3.1. Test Materials and Instruments

**Figure 9:**
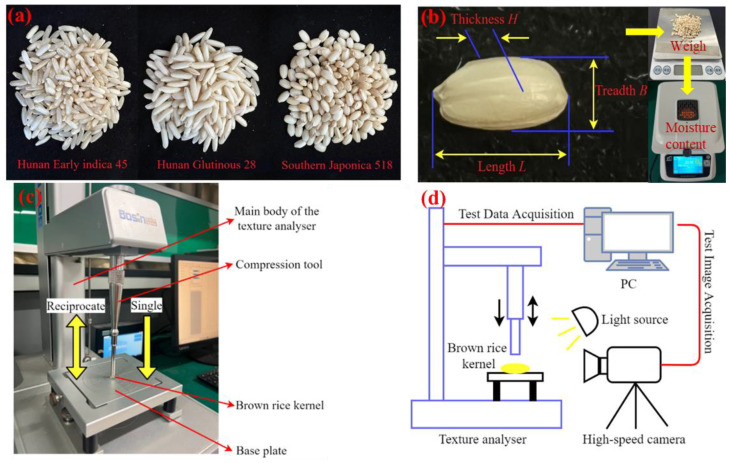
Materials and methods. (**a**) Varieties of experimental brown rice kernels; (**b**) pretreatment of materials; (**c**) single and continuous compression tests; (**d**) test schematic.

The test materials selected for this study were as follows: Hunan Early indica 45 produced by Hunan Dongting Gaoke Seed Industry, Yueyang, China, Hunan Glutinous 28 produced by Hunan Xiangsui Seed Industry, Changsha, China, and Southern Japonica 518 produced by Jiangsu Ruihua Seed Industry, Suqian, China. Referring to the national standard [35], the moisture content of the test samples was measured as 11.28%, 12.06%, and 14.21%, the distribution of the brown rice kernels’ grain size (thickness) was divided into 1.71–1.99 mm, 1.76–2.08 mm, and 2.12–2.39 mm, and the thousand-kernel weight of the brown rice kernels was 23.84 g, 21.04 g, and 21.76 g. The varieties of experimental brown rice kernels are shown in Figure 9a, and the pretreatment of materials is shown in Figure 9b.

The test instruments we used were as follows: vernier callipers (precision 0.01 mm) for measuring parameters such as the triaxial dimensions of brown rice kernels; electronic weigher (precision 0.01 g); XF-800MB moisture detector (precision 0.01%); TA.XTC-18 type texture analyser (detection error less than 0.015%) for measuring the compression characterisation of the test materials; Windows 10 system PC; HTGE34GC/M high-speed camera; and R2890/LED ring light source.

### 3.2. Test Methods

#### 3.2.1. Single Compression Test

The steps for the single compression test include sampling, preprocessing, test parameter setting, compression test, image acquisition, and postprocessing. From each of the above three types of brown rice, 25 kernels with a complete shape, uniform grain size, and no obvious chalkiness were randomly selected as the test samples. The triaxial dimensions of all the kernels in the samples were measured using vernier callipers, and the kernels of each variety were divided into three categories using grain size (thickness) as the criterion. According to the distribution of the grain size of the brown rice kernels of the three varieties, the thicknesses of 80% of the brown rice kernels of Hunan Early indica 45, Hunan Glutinous 28, and Southern Japonica 518 were in the ranges of 1.75 mm~1.95 mm, 1.80 mm~2.00 mm, and 2.15 mm~2.35 mm, respectively. The indenter model used was TA/2, and the pretest speed was set to 60 mm/min, the test loading speed was set to 1.2 mm/min, the post-test speed was set to 60 mm/min, the loading distance was set to 1 mm, and the trigger force was set to 0.1 N. Load tests were carried out using compression deformation and the loads of different varieties of kernels when they were compressed to produce crushing as indexes, and a high-speed camera was used to record the crushing process of brown rice kernels. We derived and plotted the curve of the compression force versus the shape variable during the whole process from the beginning of compression to the complete crushing of the kernels. The single compression test and test schematic are shown in Figure 9c,d.

#### 3.2.2. Continuous Compression Test

The test procedure for continuous compression is the same as outlined above. From each of the above three types of brown rice, 740 kernels with good apparent quality and thickness in the 80% concentration distribution interval were randomly selected as the test samples. The results of the single compression test showed that the average deformation of the three types of brown rice kernels at crushing was less than 0.80 mm, 0.70 mm, and 0.80 mm for Hunan Early indica 45, Hunan Glutinous 28, and Southern Japonica 518, respectively. To ensure the accuracy of the test, the loading distance set for the test did not exceed the crushing average deformation. The compression tool model used was TA/2, and we set the pretest speed to 60 mm/min, the test loading speed to 1.2 mm/min, and the post-test speed to 60 mm/min. The loading distances of Hunan Early indica 45 were 0.20 mm, 0.25 mm, 0.30 mm, 0.35 mm, 0.40 mm, 0.45 mm, 0.50 mm, 0.55 mm, 0.60 mm, 0.65 mm, 0.70 mm, 0.75 mm, and 0.80 mm; the loading distances of Hunan Glutinous 28 were 0.20 mm, 0.25 mm, 0.30 mm, 0.35 mm, 0.40 mm, 0.45 mm, 0.50 mm, 0.55 mm, 0.60 mm, 0.65 mm, and 0.70 mm; and those of Southern Japonica 518 were 0.20 mm, 0.20 mm, 0.30 mm, 0.35 mm, 0.40 mm, 0.45 mm, 0.50 mm, 0.55 mm, 0.60 mm, 0.65 mm, and 0.70 mm. Multiple loading tests were carried out using the amount of compression deformation, load, and number of compressions when the kernels were subjected to continuous compression and crushing at different loading distances as indicators, and the test was repeated 20 times for the same loading distance. A high-speed camera recorded the continuous damage made to broken brown rice kernels. Compression force as a function of the shape variable was derived and plotted from initial compression to the breakage of the kernels, and the number of compression loadings until the breakage of the kernels was recorded (if more than 30 loadings were made and the kernels did not break, brown rice was considered to be free of damage). The continuous compression test and test schematic are shown in Figure 9c,d.

## 4. Results and Discussion

### 4.1. Single Compression Test Result

**Figure 10:**
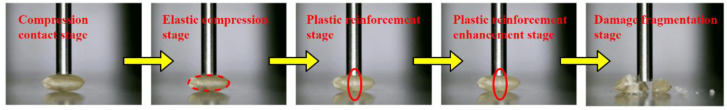
Single compression process diagram.

In this study, the single compression crushing test was conducted on three varieties of brown rice kernels: Hunan Early indica 45, Hunan Glutinous 28, and Southern Japonica 518. The mean value of the single compression force of 25 brown rice kernels of the same variety and size range, respectively, was taken, and the single compression curves of the brown rice kernels were obtained by plotting. The results of the single compression test of brown rice kernels show that the overall trend of the relationship between the compression shape variable and compression force in the single compression is basically the same. When combined with the image of the whole process of compression to crushing, the single compression process can be divided into the compression contact stage, elastic compression stage, plastic reinforcement stage, plastic reinforcement enhancement stage, and damage fragmentation stage, as shown in Figure 10.

From Equations (13) and (21), it can be seen that *F*_s_-*α*_s_^3/2^ denotes a linear relationship. We took 25 samples of three different varieties of brown rice kernels of the front end of the approximate linear part of the value of the substitution of Equation (17) and took the average value of the results of the calculations to obtain the varieties of brown rice kernels of the apparent elastic modulus *E*. Similarly, we took the three-axis dimensions of each sample and substituted them into Equation (16), and we checked the table to obtain the *k* value of each variety of brown rice kernels after taking the mean value of the calculated results. The equivalent radius *R*_e_ of each variety of brown rice kernels can be obtained by taking the three-axis dimensions of each sample and substituting them into Equation (11) and Equation (15) and averaging the calculated results. Referring to the relevant literature, the Poisson’s ratio *µ* of each variety of brown rice is 0.3 by default, and the apparent modulus of elasticity *E* of each variety of brown rice obtained from the foregoing can be substituted into Equation (14) to obtain the integrated modulus of elasticity of brown rice kernels of each variety E*. The calculation results are shown in Table 1.

Substituting the data from Table 1 into Equation (13) yields the theoretical Hertz contact curves for each variety of brown rice kernels as follows:

(26) $$\left\{\begin{array}{c} F_{indica}=529.75\delta^{\frac{3}{2}}\\ F_{japonica}=451.71\delta^{\frac{3}{2}}\\ F_{glutinous}=499.39\delta^{\frac{3}{2}} \end{array}\right.$$

### 4.2. Analysis of Single Compression Characteristics

**Figure 11:**
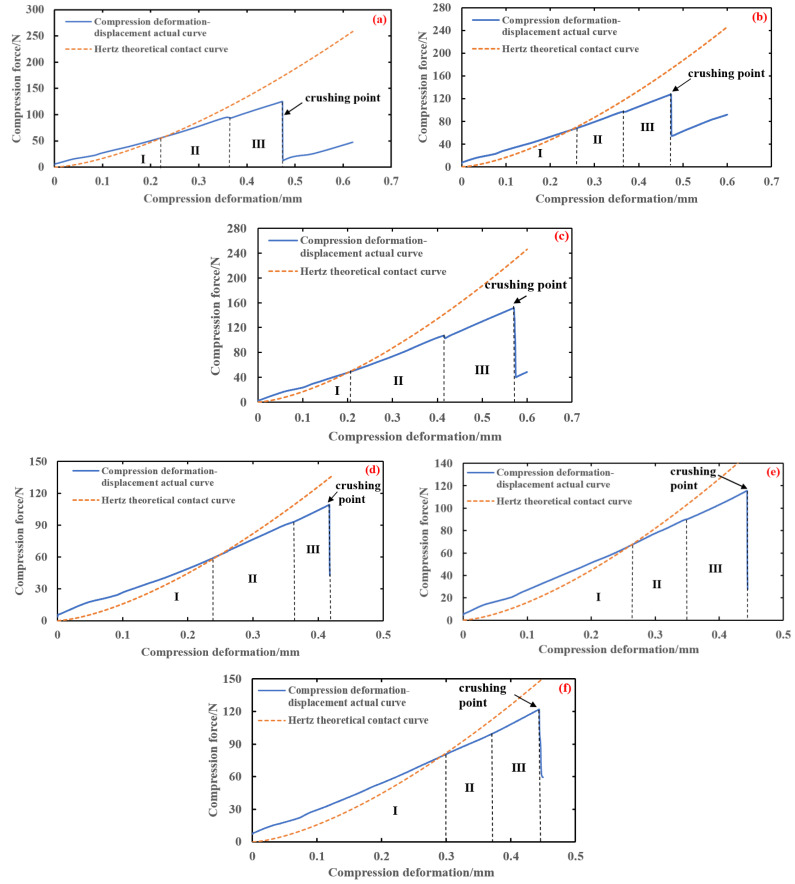

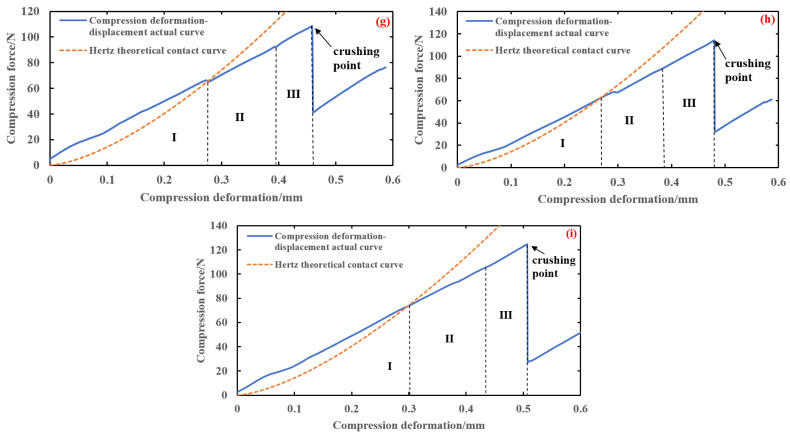
Single compression curve for brown rice. (**a**) Hunan Early indica 45—thickness 1.78 mm; (**b**) Hunan Early indica 45—thickness 1.86 mm; (**c**) Hunan Early indica 45—thickness 1.90 mm; (**d**) Hunan Glutinous 28—thickness 1.78 mm; (**e**) Hunan Glutinous 28—thickness 1.83 mm; (**f**) Hunan Glutinous 28—thickness 1.90 mm; (**g**) Southern Japonica 518—thickness 2.14 mm; (**h**) Southern Japonica 518—thickness 2.18 mm; (**i**) Southern Japonica 518—thickness 2.22 mm.

The single compression test curves of brown rice kernels and the Hertz contact theory curves are shown in Figure 11, in which all varieties of brown rice kernels do not have a significant biological yield point during compression and do not fully conform to their Hertz contact theory curves. The compression curve of brown rice kernels can be divided into three stages: the elastic compression stage (I), plastic reinforcement stage (II), and plastic reinforcement enhancement stage (III). The compression curves basically coincide with the Hertz contact theory curves at stage I, and the compression curves gradually deviate from the Hertz contact theory curves at stages II and III. The reason for this phenomenon is that the brown rice kernels are not homogeneous elastomers and therefore do not satisfy the prerequisite assumptions of the Hertz contact theory. Due to the existence of microscopic defects such as pores inside brown rice kernels, their internal structure changes with the increase in compression load, and when the compression load exceeds the elasticity threshold, brown rice produces the phenomenon of irrecoverable plastic deformation. During the first stage of compression, the brown rice kernels can be considered elastomers, which return to their original form when the compression load is removed. When the compression load exceeds the elastic critical value (the value of the vertical coordinate of the intersection of the compression test curve and the Hertz contact theory curve), it enters into the second stage of compression; at this time, the internal microscopic defects of the brown rice kernels begin to produce expansion and fusion, the brown rice kernels appear to be plastically strengthened, and their mechanical properties change. As the compression load continues to increase, the internal pores of brown rice shrink to their limit during the third stage of compression, and the change in its internal structure tends to stabilise, at which time the plastic strengthening process of brown rice kernels occurs.

The analysis shows that brown rice kernels cannot be treated as uniformly isotropic elastomers during mechanical compression but rather as elastic–plastic bodies with microscopic defects. When the load does not exceed the critical value of elasticity, the brown rice kernels can be treated as elastomers and conform to the premise assumptions of the Hertz contact theory, and their compression curves are basically in agreement with the Hertz contact theory curves. Above the elasticity threshold, the brown rice kernels undergo plastic deformation, and their compression curves begin to gradually deviate from the Hertz contact theory curves, generally showing a nearly linear plastic strengthening process.

The data from the single compression test of brown rice kernels were collated, as shown in Table 2. In particular, the maximum destructive forces *F*_c_ in the single compression of each variety of brown rice kernels were 134.77 ± 11.20 N, 115.64 ± 4.35 N, and 115.84 ± 5.89 N, respectively, and the maximum amounts of broken deformation in single compression *α*_c_ were 0.51 ± 0.04 mm, 0.43 ± 0.01 mm, and 0.48 ± 0.17 mm, respectively. The critical mean shape variables of the elastic–plastic deformation *α*_s_ were 0.224 mm, 0.267 mm, and 0.280 mm, respectively.

### 4.3. Validation of Elastic–Plastic Compression Models

In order to verify the accuracy of the elastic–plastic model of brown rice kernels, according to the known conditions in Table 3, the elastic–plastic compression model curves of the brown rice kernels of Hunan Early indica 45, Hunan Glutinous 28, and Southern Japonica 518 varieties were computed and plotted using Equation (21), and the actual compression curves of the three varieties of brown rice kernels were analysed in comparison with the curves of their Hertz contact model.

**Figure 12:**
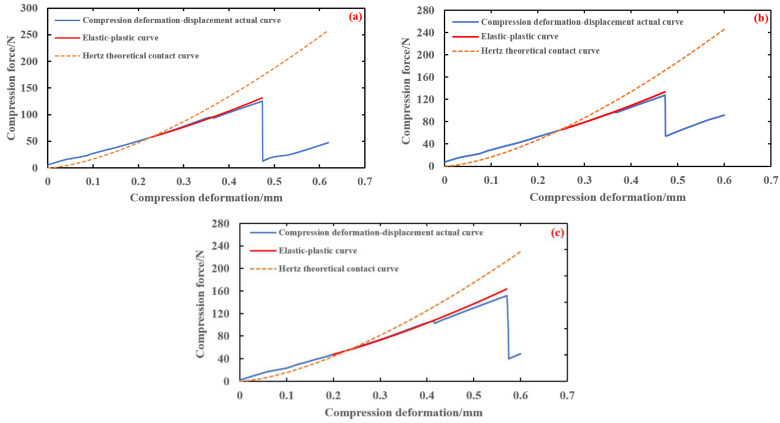

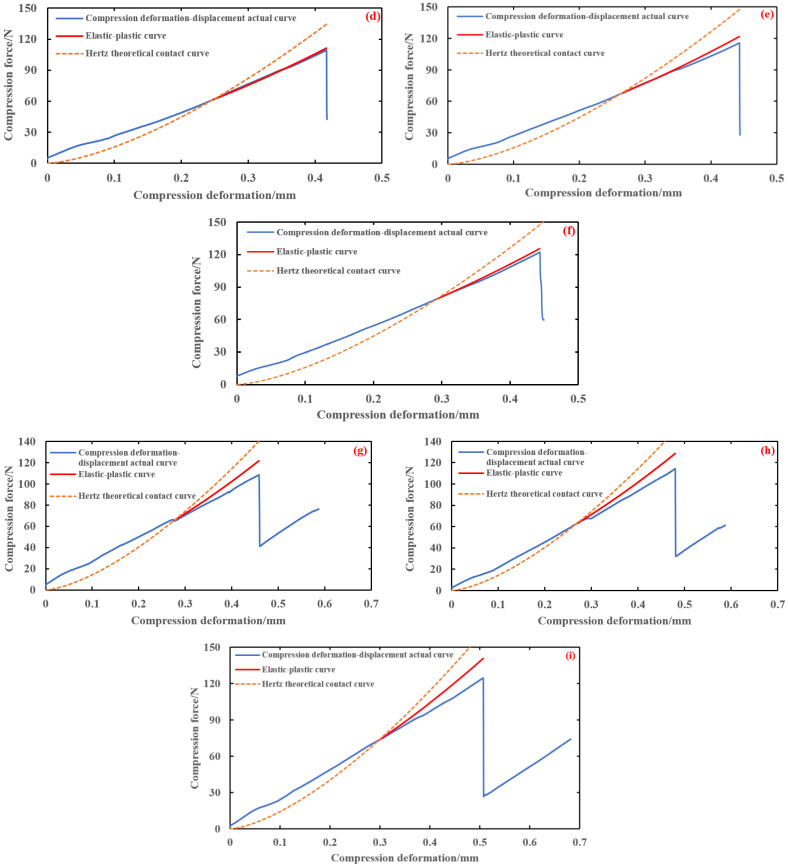
Single compression elastic–plastic curve of brown rice. (**a**) Hunan Early indica 45—thickness 1.78 mm; (**b**) Hunan Early indica 45—thickness 1.86 mm; (**c**) Hunan Early indica 45—thickness 1.90 mm; (**d**) Hunan Glutinous 28—thickness 1.78 mm; (**e**) Hunan Glutinous 28—thickness 1.83 mm; (**f**) Hunan Glutinous 28—thickness 1.90 mm; (**g**) Southern Japonica 518—thickness 2.14 mm; (**h**) Southern Japonica 518—thickness 2.18 mm; (**i**) Southern Japonica 518—thickness 2.18 mm.

As can be seen from Figure 12, the three curves intersect in the middle part, and the horizontal coordinate of the intersection point is the elastoplastic critical shape variable *α*_s_. When the compression variant is less than *α*_s_, the theoretical contact curve of Hertz and the actual compression curve are basically in good agreement. When the compression variant is more than *α*_s_, the theoretical contact curve of Hertz gradually deviates from the actual compression curve as the compression variant increases; at this time, the elastic–plastic curve and the actual compression curve are in better agreement. Therefore, the elastic–plastic model can be used to correct the curve after *α*_s_ to fit the actual compression curve more accurately. In summary, the elastic–plastic contact model proposed in this paper can better predict the relationship between compression deformation and compression force during the actual compression of brown rice kernels.

### 4.4. Continuous Compression Test Results

Neglecting the effect of the thickness of brown rice kernels, the data of the experimental results are shown in Table 4. The initial morphology intervals of Hunan Early indica 45, Hunan Glutinous 28, and Southern Japonica 518 were 0 mm~0.20 mm, 0 mm~0.25 mm, and 0 mm~0.25 mm, respectively, and applying 30 consecutive mechanical compressions to brown rice kernels within the intervals did not lead to kernel fragmentation. The continuous application of compressive load resulted in the kernels being crushed when the shape variable interval increased from 0.25 mm to 0.55 mm, 0.30 mm to 0.45 mm, and 0.30 mm to 0.50 mm, respectively, and the number of consecutively applied loads decreased with the increase in the shape variable. A single compressive load directly led to the crushing of kernels when the shape variable exceeded 0.55 mm, 0.45 mm, and 0.50 mm, respectively.

**Figure 13:**
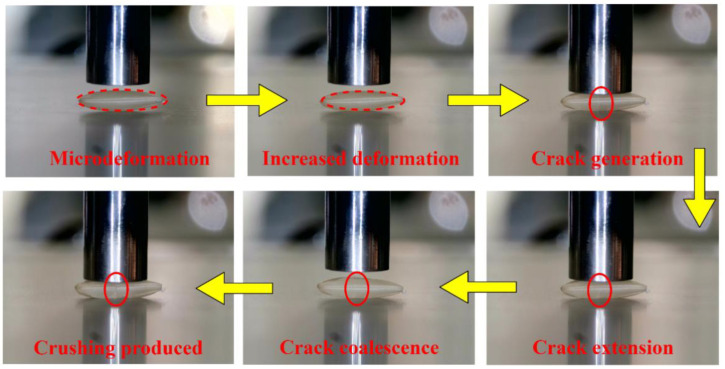
Continuous compression process.

As shown in Table 4, the damage limits of the three types of brown rice kernels were within the intervals of 0.20 mm to 0.25 mm, 0.25 mm to 0.30 mm, and 0.25 mm to 0.30 mm, respectively. The average critical values of the elastic deformation of the three types of brown rice kernels obtained from a single compression test were 0.224 mm, 0.267 mm, and 0.280 mm, respectively, which were in line with the range interval of the above damage limits. Therefore, we found that the deformation variable *α*_s_ at the time of the elastic–plastic deformation transformation of brown rice kernels is the damage limit deformation variable *α*_d_ of brown rice kernels. By combining the crack extension images of the kernels at the end of each compression during the test (Figure 13) and the previous results, it can be seen that there are microscopic defects in the brown rice kernels, and the load during mechanical compression that exceeds the damage limit leads to the transformation of the elastic–plastic deformation of the kernels, resulting in cracks in the internal part of the kernels (mainly the endosperm tissues). Additionally, the successive application of compression load will promote the accumulation, expansion, and fusion of cracks, which ultimately leads to the crushing of the kernels.

### 4.5. Analysis of Continuous Compression Characteristics

From the results of the continuous compression of brown rice kernels, it can be seen that the continuous application of compression load in the initial deformation interval does not lead to kernel crushing, but the continuous application of compression load in a certain deformation interval after increasing the deformation variable does lead to kernel crushing, and a single compression load when the deformation variable exceeds the limiting value can directly lead to the crushing of the kernels.

Using Hunan Glutinous 28 brown rice kernels as an example, we used six continuous compressions to produce crushing, and the compression deformation was 0.35 mm of the experimental results of continuous damage compression force with the deformation change curve. We recorded each compression of the brown rice kernels at the time of the characteristics of the numerical value and drew a table, and then the curve and the table were analysed to explore the continuous compression process of the damage law and the evolution of the process. As can be seen from Table 5, the critical shape variables of the damage formation *α*_d_ of Hunan Glutinous 28 brown rice kernels at each compression number were 0.25, 0.26, 0.27, 0.26, 0.25, and 0.23, and their mean value was 0.253, which was in line with the range interval of the previously mentioned damage limit of 0.25 mm to 0.30 mm.

**Figure 14:**
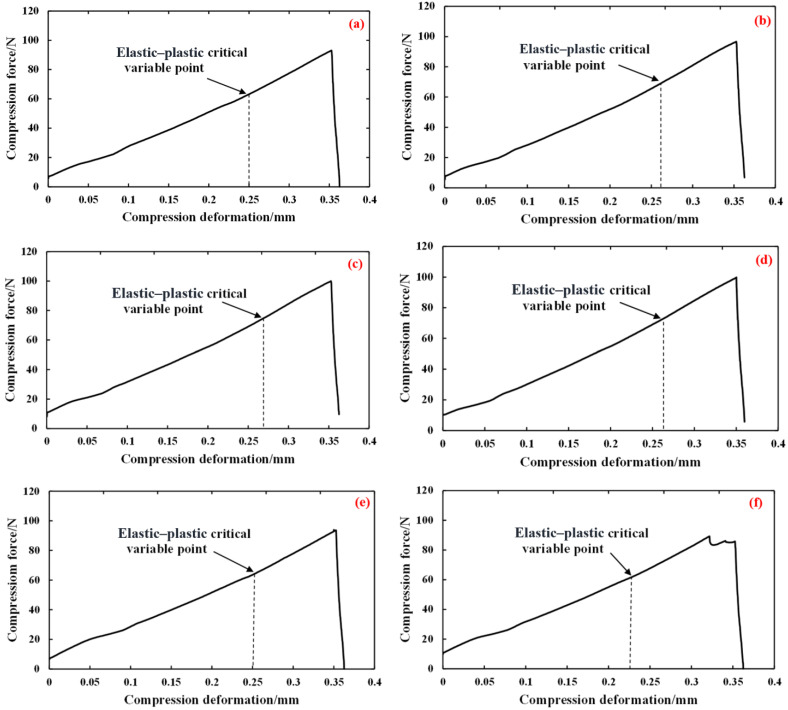
The 0.35 mm Hunan Glutinous 28 continuous compression curve. (**a**) First compression; (**b**) second compression; (**c**) third compression; (**d**) fourth compression; (**e**) fifth compression; (**f**) sixth compression.

As shown by the curves in Figure 14 and the data in Table 5, the *F*-*α_d_*^3/2^ ratio of brown rice kernels tended to increase gradually with the increase in the number of compressions. Referring to the theory of continuous damage described in the previous section, the reason for this increase is that the E*R^1/2^ of the nth compression relative to the *n* − 1st compression of the kernels increases, and it can be seen from Equation (24) that the continuous compression produces an accumulation of damage within the kernels, which leads to a decline in the mechanical properties of the kernels, numerically manifesting as a decrease in the integrated elastic modulus E*. When compression deformation exceeds the critical value of elastic–plastic deformation, the kernels become unrecoverable, and since the ratio of the unrecoverable deformation of brown rice kernels to the thickness of the kernels is large and non-negligible, there is a significant change in the equivalent contact radius of the kernels during continuous compression, and the equivalent contact radius of the kernels, Re, changes significantly. In summary, E*R^1/2^ increases as the number of compressions *n* increases, where E* is decreasing and *R*_e_ is increasing.

### 4.6. Validation of Continuous Damage Models

**Figure 15:**
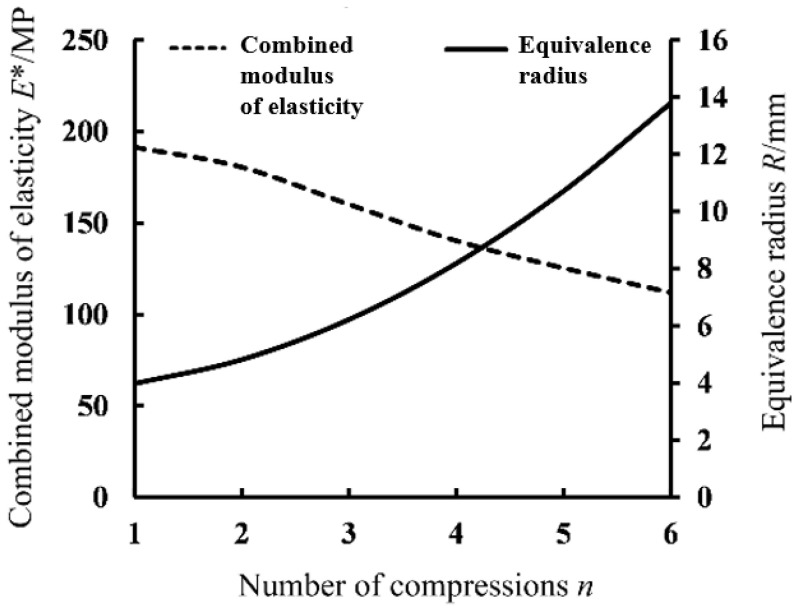
Curves of the variation in the integrated modulus of elasticity and the equivalent radius.

Figure 15 shows the variation curves of the integrated modulus of elasticity and equivalent radius with the number of compressions, calculated from the continuous compression tests. From the figure, it can be seen that the integrated modulus of elasticity of brown rice kernels decreases gradually with the increase in the number of compressions, and the sixth compression produces crushing, at which time the integrated modulus of elasticity decreases to 1/2 of the initial value. The equivalent radius of the brown rice kernels increases with the number of compressions, and the relationship shows an approximate quadratic incremental curve, with the sixth compression yielding crushing, at which point the equivalent radius increases to 7/2 of the initial value. The aforementioned theoretical analyses and the continuum damage model are consistent with the experimental results, indicating that the continuous compressive damage evolution process of brown rice kernels satisfies the linear cumulative damage criterion proposed in the continuum damage theory.

## 5. Conclusions and Outlook

The following conclusions can be drawn:

1. In this study, an elastic–plastic model of brown rice kernels is established, and equations are derived for the relationship between compression force and deformation in the elastic–plastic compression process of brown rice kernels.
2. The results of the single compression and crushing test of brown rice kernels showed that the maximum compression forces *F*_c_ of Hunan Early indica 45, Hunan Glutinous 28, and Southern Japonica 518 kernels were 134.77 ± 11.20 N, 115.64 ± 4.35 N, and 115.84 ± 5.89 N, respectively; the maximum deformation amounts were *α*_c_ 0.51 ± 0.04 mm, 0.43 ± 0.01 mm, and 0.48 ± 0.17 mm, respectively; and the average critical values of elastic–plastic deformation *α*_s_ were 0.224 mm, 0.267 mm, and 0.280 mm, respectively.
3. In this study, equations were developed for the evolution of compression damage and the cumulation of continuous compression damage of brown rice kernels, as well as the relationship between compression force and deformation under continuous compression.
4. The results of the continuous compression crushing test on brown rice kernels revealed that the critical shape variables *α*_d_ that produce damage were 0.224 mm, 0.267 mm, and 0.280 mm, respectively, and the ratios *δ* of the critical shape variables of damage to the thickness of the kernels were 12.24%, 14.35%, and 12.84%, respectively.
5. When the compression shape variable is less than *α*_d_, the continuous application of compression load does not lead to the crushing of kernels; when the compression shape variable is in the range of *α*_d_–*α*_c_, the continuous application of compression load does not lead to the crushing of kernels, and the larger the compression shape variable, the fewer the number of compressions that are required for crushing; and when the compression shape variable is more than *α*_c_, a single compression load can directly cause kernels to break. Therefore, according to the variety of brown rice, to prevent brown rice deformation, the load should be less than *α*_d_, and the maximum load should not exceed *F*_c_.
6. During the single and continuous compression testing of brown rice kernels, it is difficult to directly observe the stages of elastic–plastic changes and the generation, expansion, and fusion of continuous damage cracks at a macroscopic level. The accuracy of the model could be further intuitively verified if the structural and defective changes within the kernels during the testing process could be subsequently processed via microscopic recording. In addition, dynamic changes occur in the compression contact orientation of brown rice kernels during the milling process, and there are also differences in the moisture content of brown rice kernels. In the future, we aim to continue to combine the elastic–plastic contact theory with the theory of continuous damage to carry out analyses of the damage and crushing characteristics of brown rice kernels under different contact orientations and moisture contents, as well as elucidate the relationship between individual and groups of brown rice kernels under mechanical action, thus providing theoretical references for the damage and crushing mechanisms produced by mechanical action on brown rice.

## Figures and Tables

**Table 1:** Parameters of mechanical compression equation for brown rice kernels.

Varieties	*k*	Poisson’s Ratio *µ*	Equivalence Radius *R*_e_/mm	Apparent Modulus of Elasticity *E*/N·mm^−2^	Combined Modulus of Elasticity E*/N·mm^−2^
Hunan Early indica 45	0.851	0.3	4.07	178.99	196.69
Hunan Glutinous 28	0.910	0.3	3.98	170.84	187.74
Southern Japonica 518	0.796	0.3	3.44	166.22	182.66

**Table 2:** Single compression test data for brown rice kernels.

Varieties	Average Diameter *R*’/mm	Average Maximum Crushing force *F*_c_/N	Average Maximum Broken Shape Variable *α*_c_/mm	Average Critical Value of Elastic–Plastic Deformation *α*_s_/mm	Average Critical Value of Elastic–Plastic Deformation/Thickness of Kernels *δ/%*
Hunan Early indica 45	1.83 ± 0.09	134.77 ± 11.20	0.51 ± 0.04	0.224	12.24
Hunan Glutinous 28	1.86 ± 0.01	115.64 ± 4.35	0.43 ± 0.01	0.267	14.35
Southern Japonica 518	2.18 ± 0.03	115.84 ± 5.89	0.48 ± 0.17	0.280	12.84

**Table 3:** Test data on the elastic–plastic compression stage of brown rice kernels.

Varieties	Thicknesses *T*/mm	Critical Compression Force *F*_c_/N	Critical Deformation *α*_s_/mm
Hunan Early indica 45	1.78	125.15	0.227
	1.86	127.60	0.245
	1.90	151.56	0.200
Hunan Glutinous 28	1.78	109.24	0.248
	1.83	115.52	0.261
	1.90	122.17	0.291
Southern Japonica 518	2.14	108.58	0.276
	2.18	114.29	0.267
	2.22	124.71	0.297

**Table 4:** Results of multiple compression crushing of brown rice kernels.

Compression Deformation α_d_/mm		0.20	0.25	0.30	0.35	0.40	0.45	0.50	0.55	0.60
Hunan Early indica 45	Average number of compressions	>30	28.6	13.7	8.6	6.1	4.3	1.5	1.3	1.0
Hunan Glutinous 28		>30	>30	21.8	6.8	4.2	1.5	1.0	1.0	1.0
Southern Japonica 518		>30	>30	26.3	14.5	5.2	3.1	1.6	1.0	1.0

**Table 5:** Results of continuous compression test of brown rice kernels of Hunan Glutinous 28.

Number of Compressions	1	2	3	4	5	6
Elastic–plastic critical force *F/*N	63.67	70.09	74.78	70.97	68.40	61.15
Elastic–plastic critical variables *α_d_/*mm	0.25	0.26	0.27	0.26	0.25	0.23
*F/α_d_^3/2^*	509.36	528.68	533.02	535.32	547.20	554.38
Equivalence radius *R_e_*/mm	3.98	4.76	6.53	8.45	10.69	13.98
Combined modulus of elasticity E*/N·mm^−2^	191.49	180.61	160.29	140.38	125.46	112.01

## Data Availability

The original contributions presented in the study are included in the article, further inquiries can be directed to the corresponding author.
